# Editorial: Developments and new insights in coronary physiology and microvascular disease

**DOI:** 10.3389/fcvm.2023.1358827

**Published:** 2023-01-24

**Authors:** James Cotton, Lampson Fan

**Affiliations:** ^1^ Royal Wolverhampton Hospitals NHS Trust, Wolverhampton, United Kingdom; ^2^ University of Wolverhampton, Wolverhampton, United Kingdom

**Keywords:** coronary physiology, microvascular dysfunction, angina, coronary artery, microvascular resistance, pressure wire

**Editorial on the Research Topic** Developments and new insights in coronary physiology and microvascular disease

Our understanding of the pathophysiology of ischaemia related chest pain has been revolutionised by the development of techniques that can assess both coronary and cardiac microvascular function. Not only are these techniques rapidly becoming adopted in routine practice, they are informing our entire approach to patients with hitherto difficult to understand symptoms. We can now provide diagnoses and treatment for groups of patients poorly served by standard angiography-based assessment, particularly female patients with atypical ischaemic symptoms, those with concurrent myocardial disease and patients with chest pain and associated heart failure with a preserved ejection fraction. Whilst we are rapidly learning how to apply this current technology, a number of important therapeutic questions remain.

In this focussed issue, the included papers aim to further our understanding of this exciting topic.

In the first manuscript, Thosar et al. describe a fascinating and possibly crucial phenomenon—the diurnal variation seen in resting coronary microvascular flow and the impact that his might have on the measurement of microvascular flow reserve in practise. They postulate that the differences in microvascular tone dependant on the time of day might, in part, be responsible for the preponderance of symptoms and events seen in the morning in patients' coronary microvascular dysfunction. This retrospective echocardiographic analysis, showing raised microvascular tone in the mornings, might have important implications for our understanding of this complex set of conditions and inform future trial work.

The second manuscript in this issue continues the theme of challenging our preconceptions about the pathophysiology and assessment of ischaemia with no obstructive coronary disease (INOCA). Milzi et al. detail the results of a fascinating study assessing the utility of angiographically derived index of microvascular resistance (aIMR). They provide confirmatory evidence that this exciting technique, solely using the already available coronary angiogram data to assess microvascular function, can detect clinically important INOCA. They show that the technique yields raised aIMR levels in those with stress cMRI confirmed reversible ischaemia without epicardial stenosis when compared to patients with epicardial stenosis and demonstrated ischaemia and those without either ischaemia or epicardial stenosis. Intriguingly, they also show that the aIMR readings are raised in coronary arteries supplying the geographic myocardial territory of ischaemia demonstrated on cMRI. Current invasive assessment of IMR often focusses on one artery (typically the LAD) with the (usually correct) assumption that CMD is a generalised microvascular disorder. This paper challenges this hypothesis and also suggests a possible parallel pathophysiological process that might be localised or coronary specific.

Further putative insights are advanced by Taylor et al. who present findings indicating that female patients have a higher resting coronary microvascular resistance (CMVR) than males. They have used a computational fluid dynamics method, applied to data from coronary angiography and standard pressure wire measurements to calculate the resting CMVR in patients with significant epicardial stenosis. Their findings might, in part, help to explain the gender disparity seen in patients suffering from the symptoms of coronary microvascular dysfunction and provide a foundation for future studies. The authors also provide a practical demonstration of the value of a computational fluid dynamics technique, which will likely prove a powerful tool for future studies.

While the interventional community learns more about the underlying mechanisms and diagnosis of INOCA, our treatment options remain limited. Important advances have been made in tailoring therapy but as yet, no definitive treatment protocols have been developed. McChord et al. provide a review of current therapy utilising current antianginal therapies and detail the ongoing trials in this area. They have performed a literature review and identified two pathways that might yield results in the treatment of INOCA: the blockade of the endothelin-1 (ET-1) receptor and the stimulation of soluble guanylate cyclase (sGC).

Finally, Alisiddiq et al. and Rehan et al. provide state of the art reviews of the current body of knowledge regarding coronary physiology, iNOCA and associated pathophysiology, investigations and treatment.

